# Rectum Protection by Rectal Gel Injection in Cervical Cancer Brachytherapy: A Dosimetric Study *via* Deformable Surface Dose Accumulation and Machine-Learning-Based Discriminative Modeling

**DOI:** 10.3389/fonc.2021.657208

**Published:** 2021-04-16

**Authors:** Xuetao Wang, Bailin Zhang, Qiang He, Yilin Kong, Zhenhui Dai, Haoyu Meng, Fangjun Huang, Shengfeng Zhang, Yuanhu Zhu, Xiang Tan, Xin Zhen

**Affiliations:** ^1^ Radiation Oncology Department, The Second Affiliated Hospital of Guangzhou University of Chinese Medicine, Guangzhou, China; ^2^ School of Biomedical Engineering, Southern Medical University, Guangzhou, China

**Keywords:** brachytherapy, cervical cancer, dose accumulation, *Kushen Ningjiao*, rectum

## Abstract

**Purpose:**

This retrospective study aimed to evaluate the dosimetric effects of a rectal insertion of *Kushen Ningjiao* on rectal protection using deformable dose accumulation and machine learning–based discriminative modelling.

**Materials and Methods:**

Sixty-two patients with cervical cancer enrolled in a clinical trial, who received a *Kushen Ningjiao* injection of 20 g into their rectum for rectal protection *via* high–dose rate brachytherapy (HDR-BT, 6 Gy/f), were studied. The cumulative equivalent 2-Gy fractional rectal surface dose was deformably summed using an in-house-developed topography-preserved point-matching deformable image registration method. The cumulative three-dimensional (3D) dose was flattened and mapped to a two-dimensional (2D) plane to obtain the rectal surface dose map (RSDM). For analysis, the rectal dose (RD) was further subdivided as follows: whole, anterior, and posterior 3D-RD and 2D-RSDM. The dose–volume parameters (DVPs) were extracted from the 3D-RD, while the dose geometric parameters (DGPs) and textures were extracted from the 2D-RSDM. These features were fed into 192 classification models (built with 8 classifiers and 24 feature selection methods) for discriminating the dose distributions between pre-*Kushen Ningjiao* and pro-*Kushen Ningjiao*.

**Results:**

The rectal insertion of *Kushen Ningjiao* dialated the rectum in the ambilateral direction, with the rectal column increased from pre-*KN* 15 cm^3^ to post-*KN* 18 cm^3^ (*P* < 0.001). The characteristics of DGPs accounted for the largest portions of the top-ranked features. The top-ranked dosimetric features extracted from the posterior rectum were more reliable indicators of the dosimetric effects/changes introduced by the rectal insertion of *Kushen Ningjiao*. A significant dosimetric impact was found on the dose–volume parameters D_1.0cc_–D_2.5cc_ extracted on the posterior rectal wall.

**Conclusions:**

The rectal insertion of *Kushen Ningjiao* incurs significant dosimetric changes on the posterior rectal wall. Whether this effect is eventually translated into clinical gains requires further long-term follow-up and more clinical data for confirmation.

## Introduction

The standard treatment regimen for locally advanced cervical cancer is external beam radiotherapy (EBRT) followed by high–dose rate brachytherapy (HDR-BT). Abundant clinical evidence endorses that the local control rate positively correlates with the increasing target dose (1). However, reducing radiation toxicity to the nearby organs at risk (OARs), for example, the rectum, bladder, and vagina, is still a priority concern for safe target dose escalation. Specifically, exposure of excessive radiation to the rectum induces telangiectases, mucosal damage, fibrosis, and ischemia on the rectal wall and ultimately develops into radiation proctitis (2–4).

Some published studies reported that displacing the OARs away from the brachytherapy source is theoretically feasible in HDR-BT, minimizing radiation-induced complications (5–11). For example, Damato et al. assessed the injection of a novel hydrogel between the cervix, rectum, and bladder in female cadavers and compared it with the standard gauze packing for OAR sparing in cervical cancer brachytherapy. The study revealed a significant decrease in rectal D_2cc_ associated with the use of hydrogel (7). Kashihara et al. used a perirectal hyaluronate gel injection in gynecologic brachytherapy, confirming its safety and effectiveness for dose reduction to the rectum (9). Rai et al. (11) compared the use of a bladder–rectum spacer balloon and standard gauze packing and found that the bladder–rectum spacer balloon helped in a statistically significant dose reduction in small high-dose volumes in the rectum. Similarly, in a previous study, we prospectively evaluated the safety and efficacy of using a novel *Kushen Ningjiao* for rectal sparing in cervical cancer brachytherapy (12). *Kushen Ningjiao* is a semisolid gel made of a mixture of matrine (C_15_H_24_N_2_O) and carbopol. Matrine is an alkaloid from the traditional Chinese herb medicine *Sophora flavescens* reported to exhibit anti-inflammatory, anti-bacterial, and protective effects on cancers (13–16). The preliminary results of fractional rectal dose analysis exhibited a significant decrease in the mean D_2cc_ in the posterior rectal wall (12).

This follow-up study intended to comprehensively investigate further the dosimetric impact of the insertion of *Kushen Ningjiao* on the rectum. Two issues considered to better understand the correlation between rectal toxicity and dose were as follows. First, large inter-fractional rectum deformations should be elucidated for estimating the accumulative dose accurately on the rectum. The current clinical practice routinely uses the worst-case addition method (assuming the hotspot is stationary across treatment fractions) to evaluate D_0.1/1/2cc_ for toxicity prediction. Overestimating the OAR dose and potentially prohibiting a higher dose prescription to the target are the drawback of this method (17). The deformable accumulative dose that compensates for inter-fractional organ variations in dose summation may reflect a more accurate dose administered to the rectum. Second, traditional dose–volume parameters and D_0.1/1/2cc_ ignore dosimetric spatial information, which may help locate radiation-sensitive regions on the rectum (17–19). In particular, the three-dimensional (3D) rectal surface dose can be mapped to a two-dimensional (2D) plane so as to generate a rectal surface dose map (RSDM). The RSDM preserves the information of the geometrical dosimetric dose and can theoretically provide more insights for correlating rectal toxicity and dose patterns.

This retrospective study analyzed the dosimetric impact of the insertion of *Kushen Ningjiao* on the rectum. The fractional rectal surface dose was first summed by an in-house-developed topography-preserved point-matching deformable image registration method. The accumulative rectal dose was mapped to a 2D RSDM, from which dose–volume parameters (DVPs), texture features, and dose geometric parameters (DGPs) were extracted. Discrimination modeling was performed to differentiate the pre- and post-*Kushen Ningjiao* insertion groups, and the top-ranked dosimetric features separating the two groups were identified.

## Materials and Methods

### Patient Cohort

**Figure 1:**
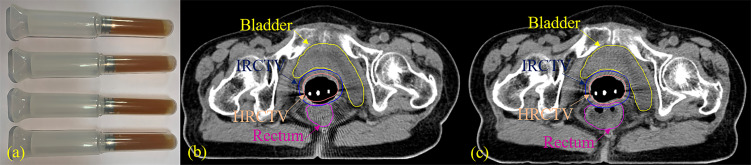
**(A)** *Kushen Ningjiao* (20 g, 5 g × 4); **(B)** pre- and **(C)** post-*Kushen Ningjiao*.

This study was approved by the institutional review board. A total of 62 patients with pathologically confirmed cervical cancer (aged from 32–83 years, mean age 56.7 years; FIGO (International Federation of Gynecology and Obstetrics) stage I–IV) treated with 25 fractional EBRT (2 Gy/f) followed by 5 fractional HDR-BT (6 Gy/f) between March 2018 and July 2019 at the Radiation Oncology Department of Guangzhou University of the Chinese Medicine Second Affiliated Hospital were retrospectively examined. The patients were enrolled in a clinical trial in which a novel rectum protection approach was used in brachytherapy. All the patients received an insertion of 20 g (5 g × 4, **Figure 1A**) of *Kushen Ningjiao* into their rectum during HDR-BT with the purpose of dilating the rectum and pushing the rectal wall away from the radiation source.

The rectal insertion of *Kushen Ningjiao* was accomplished after needle implantation using the following procedure: (1) tumor dimensions and degree of tumor extension were assessed by a gynecologic examination. Vagina and tumor were exposed by inserting a sterile speculum, which was followed by the insertion of a stainless-steel tube applicator into the uterine cavity and needle implantation into the tumor. The tube and needles were fixed with a button stopper, and the vagina was packed with gauze to prevent needle movement. Supine-position computed tomography (CT) scans were obtained as a pre-treatment and served as the comparison baseline. The CT images thus acquired were categorized as pre-*Kushen Ningjiao (pre-KN)* group. (2) Subsequently, the patient was placed in the lithotomy position, and the same attending radiation physician inserted 20 g of *Kushen Ningjiao* into the rectum. CT scanning was repeated using the same scanning protocol, and the collected CT images were classified as post-*Kushen Ningjiao (post-KN)* group. (3) Contouring was performed (by the same physician) on the pre*-KN* and post-*KN* CT images using the Oncentra treatment planning system (Nucletron, Veenendaal, The Netherlands) (**Figures 1B, C**).

For HDR-BT, the first two fractions were planned with the clinical target volume (CTV) that included the uterus plus vagina. In the following three brachytherapies, the CTV was adjusted according to the patient’s clinical condition, which was confirmed by CT/MRI imaging. A 5-mm 3D margin was used for all brachytherapy fractions. The rectum was delineated from the ischial tuberosities up to the rectosigmoid flexure.

The rectum physical doses received in HDR-BT were converted into EQD2 doses using a linear quadratic model (20) with an *α*/*β* ratio of 3 for dose summation to account for the biologic effects of different fractionation schemes (21, 22).

### Deformable Dose Accumulation and Rectum Unfolding

For each patient, the rectal wall contours in each HDR-BT fraction were converted into a mesh *via* an open-source mesh generator, *iso2mesh* (23). The fractional rectum surface meshes were registered to a reference domain (i.e., the first HDR-BT fraction) by an in-house-developed topography-preserved point-matching deformable image registration (TOP-DIR) algorithm (24). The calculated deformation vector fields were applied to deform and sum all the fractional HDR-BT doses to the first HDR-BT fraction so as to yield the final cumulative dose on the rectal wall [termed 3D rectal dose (3D-RD)].

**Figure 2:**
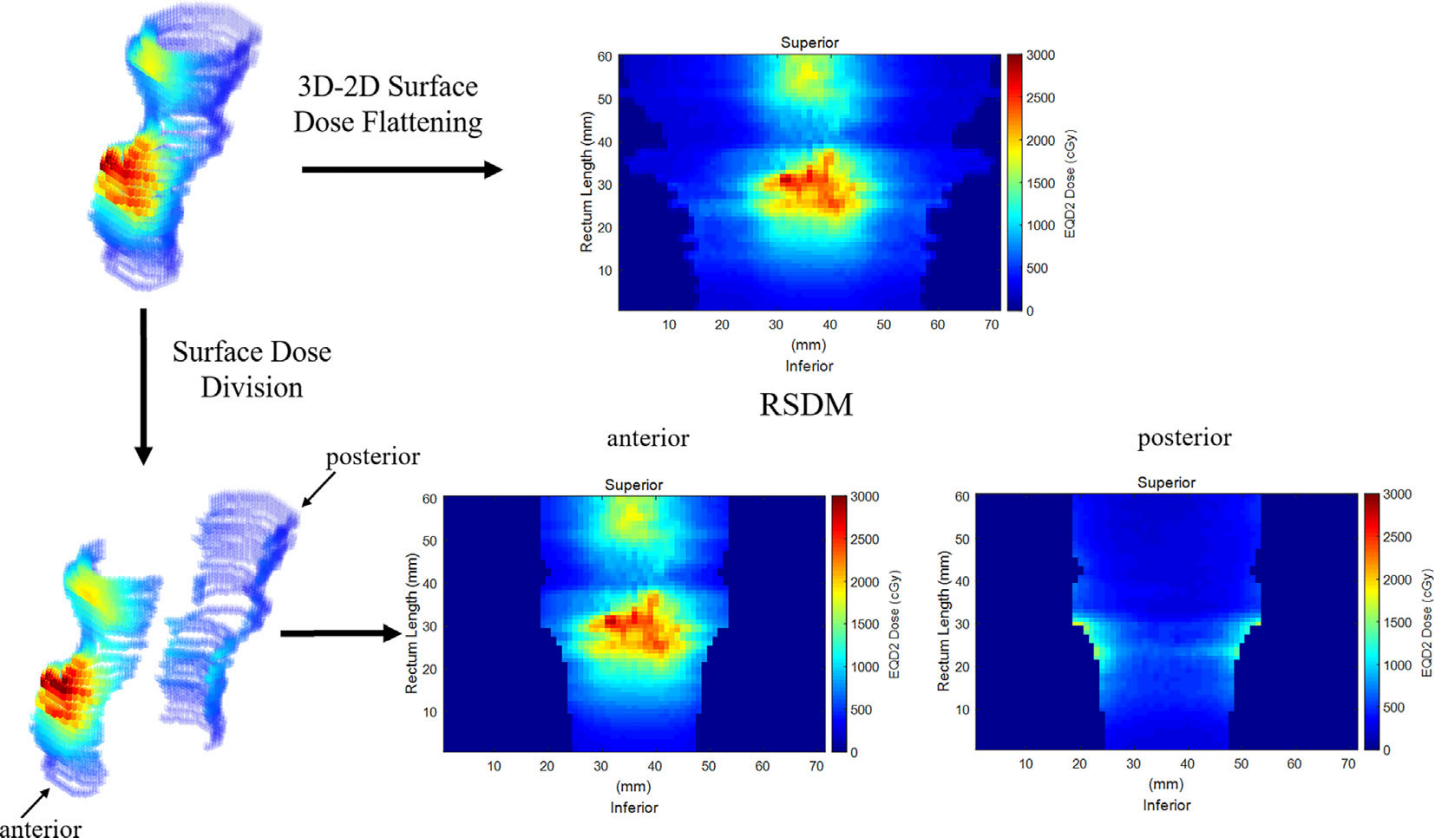
Flattening the 3D rectum surface dose to obtain 2D RSDM. The rectum was subdivided into anterior and posterior parts.

The 3D-RD was flattened and mapped onto a two-dimensional plane to obtain the 2D-RSDM *via* a mapping procedure detailed in a previous study (25). The RSDMs had a fixed image resolution (1mm × 1mm) but patient-specific image sizes (**Figure 2**) depending on the rectum circumference on each CT slice and the inferior-superior rectum length. For analytic purposes, the anterior and posterior parts of the rectum were defined based on the boundary of 50% of the rectum circumference on each slice. Hence, the rectum dose was subdivided and analyzed on the whole, anterior, and posterior 3D-RD and 2D-RSDM (**Figure 2**).

### Dosimetric Feature Extraction

Three types of dosimetric features, that is, the DVPs, texture features, and DGPs, were extracted from the whole, anterior, and posterior 3D-RD and 2D-RSDM (**Table 1**).

**Table 1:** Extracted dosimetric features.

DVPs (50)	D_0.1cc_–D_5.0cc_
**Textures** (43)	Global (*n* = 3), GLCM (*n* = 9), GLRLM (*n* = 13), GLSZM (*n* = 13), NGTDM (*n* = 5)
**DGPs** (270)	Area_1Gy–Area_30Gy, Rel_area_1Gy–Rel_area_30Gy, Ecc_1Gy–Ecc_30Gy, Maj_1Gy–Maj_30Gy, Min_1Gy–Min_30Gy, Per_1Gy–Per_30Gy, Cen2But_1Gy–Cen2But_30Gy, Cen2Lft_1Gy–Cen2Lft_30Gy, Cen2Rgt_1Gy–Cen2Rgt_30Gy

Specifically, the DVPs (50 in total) were the D*x*-cc (the minimum dose in the most exposed *x*-cm^3^ volume, *x* ∈ [0.1, 5.0] with 0.1-cm^3^ intervals) calculated from the 3D-RD.

The texture features (43 in total) were extracted from 2D-RSDM using an open-source radiomics toolbox (26), including 3 first-order gray-level statistical global features, 9 gray-level co-occurrence matrix features, 13 gray-level run-length matrix features, 13 gray-level size zone matrix features, and 5 neighborhood gray-tone difference matrix features.

**Figure 3:**
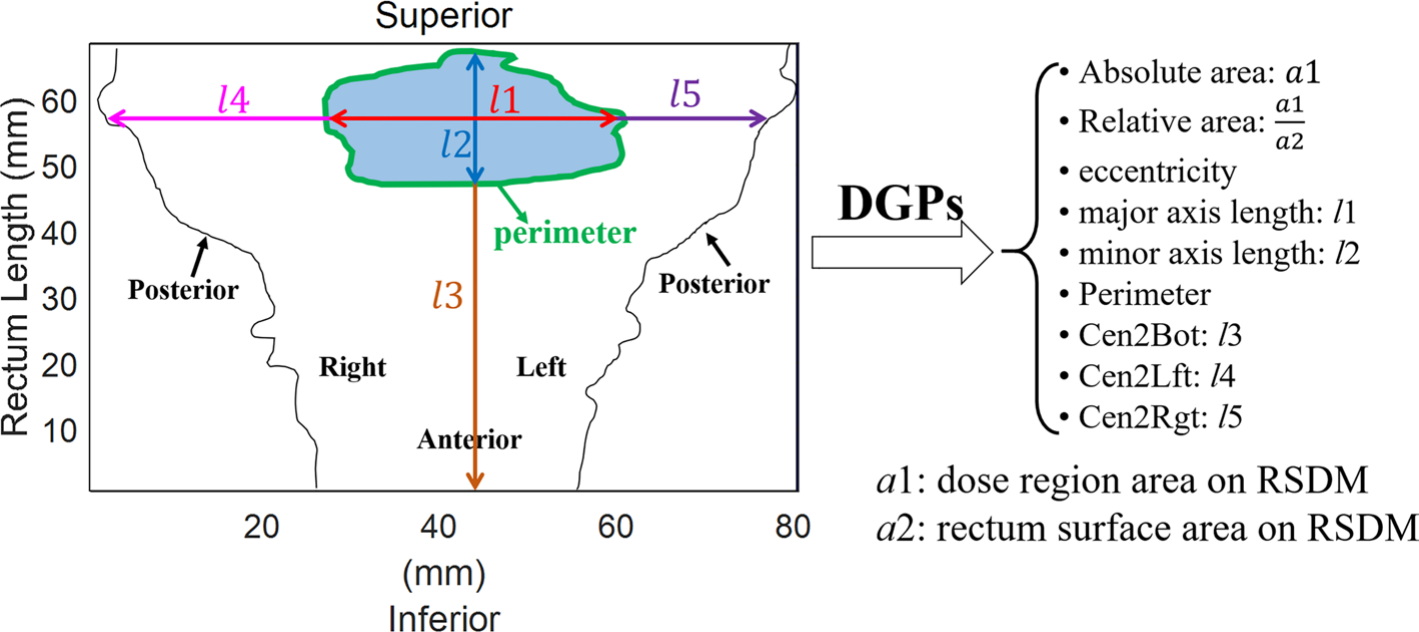
DGPs extracted from the 2D-RSDM.

Nine types of DGPs [*n* = 270 (9 × 30) in total] were computed from the 2D-RSDM at various dose levels (1−30 Gy, with a 1-Gy interval) including: (1) the absolute area (Area_1Gy–Area_30Gy, mm^2^) of a given dose level on the RSDM; which was calculated for the region with dose ≥ (or <) a specific dose level for the whole and anterior RSDM (or for the posterior rectum); (2) the relative area (Rel_area_1Gy–Rel_area_30Gy, %) of the dose region with respect to the area of the rectum surface on the RSDM; (3) the dose region eccentricity (Ecc_1Gy–Ecc_30Gy); (4) the major axis length (Maj_1Gy–Maj_30Gy, mm); (5) the minor axis length (Min_1Gy–Min30_Gy, mm); 6) the dose region perimeter (Per_1Gy–Per_30Gy, mm); (7) the distance between the centroid of the dose region and the bottom of the rectum (Cen2Bot_1Gy–Cen2Bot_30Gy, mm); (8) the distance between the centroid of the dose region and the leftmost region of the rectum (Cen2Lft_1Gy–Cen2Lft_30Gy, mm); and (9) the distance between the centroid of the dose region and the rightmost region of the rectum (Cen2Rgt_1Gy–Cen2Rgt_30Gy, mm). **Figure 3** illustrates the geometric parameter definition for the DGP calculation.

### Discrimination Modeling

The insertion of *Kushen Ningjiao* induced dose variations in the rectum. Some dosimetric features may play a dominating role in discriminating dose distributions between pre-*KN* and pro-*KN*. With the intention to determine which dosimetric features were most affected, this study identified these critical dosimetric features by building and applying discrimination models on the whole, anterior, and posterior 3D-RD and 2D-RSDM. Each discrimination model was built upon the combination of a feature selection strategy and a classifier, in which fivefold cross-validation was used to assess the discriminative performance of the model.

In each fold of the fivefold cross-validation, a subset (*n* = 20) of dosimetric features was first estimated by a specific feature selection method. These pre-screened features were further fed into a classifier to differentiate the dose distribution of the pre-*KN* versus post-*KN*. Twenty-four feature selection methods and 8 classifiers (listed in **Table 2**) were studied, and their possible combinations resulted in 192 discrimination models. The discriminative power of the model was quantified by the area under the receiver operating characteristic curve (AUC). The top-ranked dosimetric features (AUC > 0.80) among the 192 models were screened.

**Table 2:** Feature selection strategies and classifiers for discrimination modeling.

Feature selection strategies*^1^ (*n* = 24)	CIFE (27), CMIM (27), DISR (27), FCBF (28), ICAP (29), JMI (27), LCSI (27), MIFS (27), MIM (27), MRMR (27), fisher_score (30), lap_score (30), relief (31), SPEC (32), trace_ratio (33), ll_l21 (34), ls_l21 (34), MCFS (35), NDFS (36), RFS (37), UDFS (38), f_score (29), gini_index (29), t_score (29)
Classifier models*^2^ (*n* = 8)	Logistic regression, SVM, naïve Bayes, KNN, decision tree, bagging, random forest, AdaBoosting

The feature selection strategies and classifier models were implemented with the open-source machine learning toolkits *^1^scikit-feature (29) and *^2^scikit-learn (39), respectively.

### Statistical Analysis

All statistical analyses were performed using SPSS 22.0 software (SPSS Inc., IL, USA). The normality of the data distribution was assessed using the Shapiro–Wilk test. Normally distributed variables were reported as the mean ± standard deviation and compared using the paired-sample Student *t* test. Non-normally distributed variables were presented as the median (interquartile range, IQR) and compared using the Wilcoxon signed-rank test. A two-tailed significance level of *P* value <0.05 indicated a statistically significant difference.

## Results

### Rectum Geometric Changes and Dosimetric Comparisons

**Figure 4:**
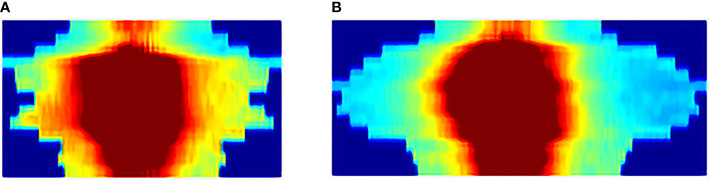
Example 2D-RSDM of a patient with **(A)** pre-*KN* and **(B)** post-*KN*.

The insertion of *Kushen Ningjiao* physically inflated the rectum, which was revealed by comparing the pre-*KN* and pro-*KN* RSDM (**Figure 4**). A longer RSDM length (or larger rectum perimeter) was observed in the pro-*KN* RSDM group than in the pre-*KN* RSDM group [138.5 (118–168.75) vs 159.5 (149.5–184) mm, *P* < 0.001]. Measuring the distance between the hottest or coldest point on the anterior or posterior rectal wall to the CTV centroid [anterior: 24.7 (23.2–26.2) vs 23.4 (22.3– 24.6) mm, *P* < 0.001; posterior: 47.1 (45.2–49.5) vs 48.8 (47.1–51.8) mm, *P* < 0.001, as shown in **Table 3**] quantitatively confirmed this point. The statistics indicated that the anterior rectal wall became closer to the CTV, and the posterior rectal wall was also pushed away from the CTV. Accordingly, the mean rectum volume was dilated from 15.0 (13.1–17.8) to 18.0 (16.4–20.3) cm^3^ (*P* < 0.001) post-*KN*. Also, the distance between the anterior and posterior rectal walls (23.3 ± 4.2 mm vs 26.5 ± 3.6 mm, *P* < 0.001) and the distance between the ambilateral rectal walls (22.7 ± 4.9 mm vs 28.1 ± 4.1 mm, *P* < 0.001) were measured. The figures showed that rectum dilation was more evident in the ambilateral direction than in the anterior–posterior direction.

**Table 3:** Rectum geometric changes and dosimetric comparisons.

			Pre-*KN*	Post-*KN*	*P* value
Distance to CTV (mm)	Anterior		24.7 (23.2–26.2)	23.4 (22.3–24.6)	<0.001^b^
	Posterior		47.1 (45.2–49.5)	48.8 (47.1–51.8)	<0.001^b^
Volume of rectal wall (cm^3^)			15.0 (13.1–17.8)	18.0 (16.4–20.3)	<0.001^b^
Distance in rectal wall (mm)	AP^1^		23.3 ± 4.2	26.5 ± 3.6	<0.001^a^
	Ambilateral		22.7 ± 4.9	28.1 ± 4.1	<0.001^a^
Dx-cc (Gy)	D_0.1cc_	Anterior	24.4 (22.9–25.9)	22.3 (20.6–24.4)	<0.001^b^
		Posterior	11.7 ± 2.2	9.6 ± 2.3	<0.001^a^
	D_1cc_	Anterior	12.7 ± 3.0	12.3 ± 2.9	=0.298^a^
		Posterior	7.5 ± 1.6	4.8 ± 1.7	<0.001^a^
	D_2cc_	Anterior	7.1 ± 3.1	7.4 ± 2.7	=0.275^a^
		Posterior	4.5 ± 2.0	2.0 ± 1.1	<0.001^a^
	D_5cc_	Anterior	1.8 (1.5–2.0)	1.8 (1.6–2.0)	=0.930^b^
		Posterior	1.2 (1.0–1.4)	0.9 (0.8–1.0)	<0.001^b^

a Paired-sample Student t test.

b Wilcoxon signed-rank test.

AP^1^, Distance between the anterior and posterior rectal walls.

Four typical dose–volume parameters, D_0.1cc_, D_1cc_, D_2cc_, and D_5cc_, are listed in **Table 3**. For the anterior rectal wall, the insertion of *Kushen Ningjiao* led to lower values of D_0.1cc_ [24.4 (22.9–25.9) vs 22.3 (20.6–24.4), *P* < 0.001] and D_1.0cc_ (12.7 ± 3.0 vs 12.3 ± 2.9, *P* = 0.298), higher values of D_2.0cc_ (7.1 ± 3.1 vs 7.4 ± 2.7, *P* = 0.275), and the same value of D_5.0cc_ [1.8 (1.5–2.0) vs 1.8 (1.6–2.0), *P* = 0.93]. For the posterior rectal wall, D_0.1cc_, D_1cc_, D_2cc_, and D_5cc_ significantly decreased after the insertion of *Kushen Ningjiao* (*P* < 0.001).

### Top-Ranked Dosimetric Features

**Figure 5:**
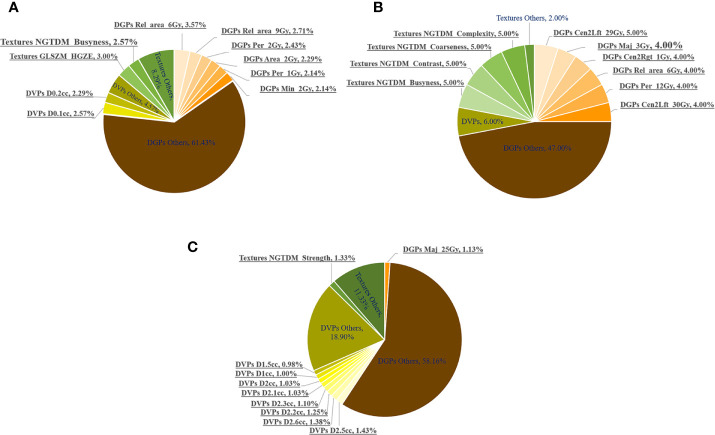
Number of times (%) the dosimetric features were selected as top 20 features in the fivefold cross-validation of all the discriminative models with AUCs >0.80. The most frequent 10 features are underlined. Number of times (%) being selected as the Top-20 features in the **(A)** whole, **(B)** anterior and **(C)** posterior rectal surface dose.

In the fivefold cross-validation, the feature selection method embedded in each discriminative model selected 20 key dosimetric features for classification. The number of each feature selected as a top-20 feature (only for models with AUC > 0.80) was determined, and the corresponding percentage was summarized, which is shown in pie charts in **Figure 5**.

DGPs accounted for the largest portion of the top-20 features, followed by DVPs and texture features. This observation was consistent for the whole, anterior, and posterior rectum. **Figure 5** highlights the following top 10 most frequently selected: (1) six DGPs, two DVPs, and two texture features for the whole rectum; (2) six DGPs and four texture features for the anterior rectum; and (3) one DGP, eight DVPs, and one texture feature for the posterior rectum.

The top 10 most frequently selected features for the whole, anterior, and posterior rectal surfaces are summarized in **Table 4**. For the whole rectum, five DGPs [Rel_area_6Gy (*P* < 0.001), Rel_area_6Gy (*P* < 0.001), Per_2Gy (*P* < 0.001), Area_2Gy (*P* < 0.001), and Per_1Gy (*P* < 0.0001)], two DVPs [D_0.1cc_ (*P* < 0.001) and D_0.2cc_ (*P* < 0.001)], and one texture feature [GLSZM_HGZE (*P* < 0.001)] were statistically significant features. These top-ranked features were mostly extracted from the low-dose regions (<10 Gy). For the anterior rectum, only three DGPs [Cen2Lft_29Gy (*P* = 0.008), Cen2Rgt_1Gy (*P* < 0.001), and Cen2Lft_30Gy (*P* = 0.029)] and one texture feature [NGTDM_Coarseness (*P* = 0.001)] were statistically significant features. For the posterior rectum, all the DVPs (D_1.0 cc~D2.6 cc_, *P* < 0.001) were statistically significant features.

**Table 4:** Top 10 most frequently selected features.

	Feature category	Top-ranked features (ranking no.)	Pre-*KN*	Post-*KN*	*P* value
Whole	DGPs (*n* = 6)	Rel_area_6Gy (1st)	55.4 ( ± 12.5)	50.5 ( ± 11.3)	<0.001^a^
		Rel_area_9Gy (3rd)	37.4 ( ± 10.4)	31.1 ( ± 8.2)	<0.001^a^
		Per_2Gy (6th)	496.9 ( ± 86.4)	527.0 ( ± 70.0)	<0.001^a^
		Area_2Gy (7th)	9,057.5 ( ± 2051.6)	10,456.6 ( ± 2062.3)	<0.001^a^
		Per_1Gy (9th)	463.7 (424.5–512.2)	516.9 (477.1–545.7)	<0.001^b^
		Min_2Gy (9th)	96.1 (84.5–106.7)	97.6 (86.9–114.4)	=0.273^b^
	DVPs (*n* = 2)	D_0.1cc_ (4th)	24.4 (22.9–25.9)	22.3 (20.6–24.4)	<0.001^b^
		D_0.2cc_ (7th)	22.1 (20.6–23.8)	20.3 (18.3–22.8)	<0.001^b^
	Textures (*n* = 2)	GLSZM_HGZE (2nd)	12,187.2 ± 3610.9	10,567.9 ± 3067.7	<0.001^a^
		NGTDM_Busyness (4th)	7.7 (6.0,9.5) (×10^-3^)	7.5 (6.5,9.5) (×10^-3^)	=0.947^b^
Anterior	DGPs (*n* = 6)	Cen2Lft_29Gy (1st)	3.1 (0.0–8.2)	0 (0.0–4.5)	=0.008^b^
		Maj_3Gy (2nd)	104.5 (89.2–120.9)	104.0 (91.7–117.5)	=0.897^b^
		Cen2Rgt_1Gy (2nd)	67.8 (57.5–82.9)	78.3 (73.3–90.5)	<0.001^b^
		Rel_area_6Gy (2nd)	36.4 ( ± 5.7)	36.3 ( ± 5.0)	=0.837^a^
		Per_12Gy (2nd)	237.4 (212.9–284.8)	230.0 (199.8–264.2)	=0.115^b^
		Cen2Lft_30Gy (2nd)	0.4 (0.0–6.7)	0.0 (0.0–1.8)	=0.029^b^
	Textures (*n* = 4)	NGTDM_Busyness (1st)	2.9 (2.4,3.8) (×10^-3^)	3.0 (2.6,3.7) (×10^-3^)	=0.864^b^
		NGTDM_Contrast (1st)	0.2 ( ± 0.08)	0.2 ( ± 0.08)	=0.713^a^
		NGTDM_Coarseness (1st)	2.1 ( ± 0.6) (×10^-2^)	1.9 ( ± 0.4) (×10^-2^)	=0.001^a^
		NGTDM_Complexity (1st)	41128.2 ( ± 11549.0)	41182.5 ( ± 11141.7)	=0.969^a^
Posterior	DGPs (*n* = 1)	Maj_25Gy (5th)	104.6 (90.2–122.4)	104.2 (92.3–120.2)	=0.952^b^
	DVPs (*n* = 8)	D_2.5cc_ (1st)	2.7 (1.9–4.6)	1.2 (1.0–1.6)	<0.001^b^
		D_2.6cc_ (2nd)	2.6 (1.8–4.4)	1.2 (1.0–1.6)	<0.001^b^
		D_2.2cc_ (4th)	3.8 (2.3–5.5)	1.4 (1.1–1.8)	<0.001^b^
		D_2.3cc_ (6th)	3.6 (2.3–5.2)	1.3 (1.1–1.8)	<0.001^b^
		D_2.1cc_ (7th)	4.2 ( ± 2.0)	1.8 ( ± 1.0)	<0.001^a^
		D_2.0cc_ (7th)	4.5 ( ± 2.0)	2.0 ( ± 1.1)	<0.001^a^
		D_1.0cc_ (9th)	7.5 ( ± 1.6)	4.8 ( ± 1.7)	<0.001^a^
		D_1.5cc_ (10th)	5.8 ( ± 1.8)	3.0 ( ± 1.5)	<0.001^a^
	Textures (*n* = 1)	NGTDM_Strength (3rd)	488.2 ( ± 136.5)	456.9 ( ± 132.3)	=0.098^a^

a Paired-sample Student t test.

b Wilcoxon signed-rank test.

### Discriminative Capability of the Top-Ranked Dosimetric Features

**Figure 6:**
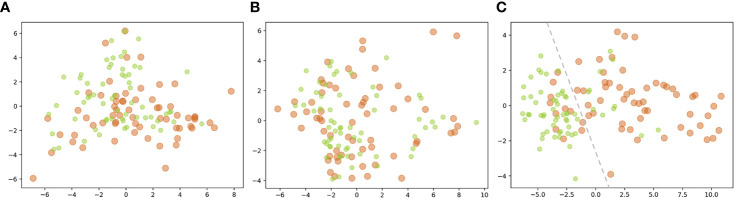
Projection of the top 10 features [**(A)** whole; **(B)** anterior; **(C)** posterior] of the pre-*KN* (green) and post-*KN* (orange) groups onto the two-dimensional scatter plot.

The isometric mapping (Isomap) method was used to visualize the top 10 features (40) by projecting the high-dimensional dataset onto a two-dimensional scatter plot (**Figure 6**). The pre-*KN* and post-*KN* groups were not discriminable using the top-ranked dosimetric features analyzed from the whole and anterior rectum. In contrast, the top-ranked dosimetric features of the posterior rectum were more effective for differentiation, as evidenced by the clear boundary (dashed lines in **Figure 6C**) separating the two groups. This result suggested that the top-ranked dosimetric features extracted from the posterior rectum were more reliable indicators for the dosimetric effects/changes introduced by the insertion of *Kushen Ningjiao*.

## Follow-ups

By the end of November 2020, the RTOG (Radiation Therapy Oncology Group) standard was used to grade eight patients who presented with rectal toxicity symptoms, confirmed *via* clinical inquiry and/or endoscopy (41). **Table 5** lists the symptoms associated with the rectal toxicity of all eight patients, among whom two patients were scored as grade 1, five patients were scored as grade 2, and one patient was scored as grade 3.

**Table 5:** Rectal toxicity grading of the eight patients who presented with symptoms.

Patient #	Symptoms	Grade
1st	Mucous bloody stool and rectal and anal ulcer	2
2nd	Diarrhea, abdominal pain, bloody mucous stool, and tenesmus	2
3rd	Bloody stool, lower gastrointestinal bleeding, and proctorrhagia	3
4th	Yellow sodden feces two to four times per day and tenesmus	1
5th	More times of stools (>5 times)	2
6th	Anal bearing-down with vague pain, tenesmus, more times of stool, and colonic polyps	2
7th	Anal bearing-down and hematochezia	2
8th	More times of stools (<5 times) and colorectal inflammation	1

## Discussion

Rectal toxicity has been extensively investigated and reported in recent studies. These studies agreed that the rectum volume receiving a high dose below a certain level (e.g., V70Gy <25%, V75Gy <5%) was predictive of a low incidence of rectal toxicity (42). Standard management for radiation proctitis is still under investigation. Current endeavors mainly explore approaches to increase the separation between the radiation source and the rectum. Currently, tissue spacers and endorectal balloons are the two main approaches. Tissue spacers are bio-absorbable spacers that physically increase the distance from the source to the rectum. Hyaluronic acid, human collagen, interstitial biodegradable balloons, and synthetic polyethylene glycol hydrogels are the main materials used for tissue spacers, as reported in the literature (43–46). Tissue spacer implantation is an invasive procedure typically performed transperineally under real-time transrectal ultrasound guidance and local, spinal, or general anesthesia. Endorectal balloons are silicon or latex devices filled with either air or water and inserted into the rectum prior to radiotherapy treatment (47). Although these balloons were initially used as immobilizers to reduce target motion (48), their dosimetric impact has also been studied. Filled balloons push the anterior rectal wall toward the high-dose exposure while increasing the distance between the source and the posterior rectal wall. Endorectal balloons significantly reduced the rectal wall volume exposed to doses >40 Gy, and decreased high-grade telangiectasias and grades 1–3 late rectal bleeding is found (49).

*Kushen Ningjiao* is similar to endorectal balloons. The rectal insertion of *Kushen Ningjiao* physically inflated the rectum and expanded the space between the posterior rectal wall and the high-dose region. As a gel, *Kushen Ningjiao* easily adapts to the shape of the rectum and is therefore estimated to incur less discomfort in retention. The insertion procedure is fast, less than 1 min, and no discomfort has been reported by the enrolled patients. The major compound matrine in *Kushen Ningjiao* is absorbable, nontoxic, and nonimmunogenic. Some pharmacologic and curative effects of matrine (anti-tumor, anti-inflammatory, and anti-bacterial effects) have been reported (13–16, 50); however, its radiobiological impact on the amelioration of rectal toxicity is still unclear. This study mainly focused on the physical benefits of *Kushen Ningjiao* by providing more protection on the posterior rectal wall. It was hypothesized that this effort would help reduce the incidence of rectal toxicity, as previous studies found correlations between rectal toxicity and dose delivered on the posterior rectal wall (51–54). For example, Dewit L et al. (52) found that the gastrointestinal radiation injury became significant when the dose on the posterior rectal wall was 65–76 Gy. Similarly, Cho et al. (51) found a statistically significant correlation (*P* < 0.005) between rectal proctitis and posterior rectal doses >50 Gy in patients with prostate cancer. More recent studies also reported consistent findings. For example, Onjukka et al. (53) analyzed the spatial pattern of dose on the anorectal wall and found that rectal bleeding was associated with high isodoses reaching the posterior rectal wall. They concluded that the pattern of sparing in the posterior rectal wall might be as important as the pattern of tissue damage in the anterior wall. Shelley et al. (54) also found statistically meaningful correlations between dose on the posterior rectal wall with rectal bleeding, proctitis, and fecal incontinence *via* voxel-level accumulated-dose analysis. However, more in-depth investigations are still required to thoroughly evaluate the dosimetric compromise between the anterior and posterior rectal walls.

Reporting accurate accumulated rectal dose over the entire treatment course is a nontrivial task because substantial inter-fractional rectum deformation exists in the HDR-BT treatments. In this study, an in-house-developed TOP-DIR method was used, which was validated to achieve accurate geometric registration accuracy on a porcine bladder phantom (~ 2 mm) (24). This organ surface point matching method was successfully applied to accumulate fractional rectal dose for rectum toxicity prediction in a previous study (25). However, deformable registration inevitably introduces uncertainties into the subsequent dosimetric analysis. Therefore, further phantom studies are still needed to quantify the geometrical registration error and the associated dosimetric impact on the summed dose.

The rectal dilation effect was evident, as the rectum volume increased from pre-*KN* 15 cm^3^ to post-*KN* 18 cm^3^ (*P <* 0.001). However, this dilation was not circularly symmetric because the dilation in the anterior–posterior direction (23.3 vs 26.5 mm) was smaller than that in the ambilateral direction (22.7 vs 28.1 mm). This difference might be attributed to the semisolid nature of the gel, which deformably conformed to the shape of the rectum and its nearby anatomies when the rectum dilated. The dominating ambilateral trend of dilation might also affect the hotspot location on the rectal wall, as seen by the drop (instead of increase) of D_0.1cc_ (24.4 vs 22.3 Gy, *P* < 0.001) and D_1cc_ (12.7 vs 12.3 Gy, *P* = 0.298) in the post-*KN*. However, the changes in D_0.1/1/2/5cc_ measured on the posterior rectal wall were consistent, and significant decreases (all *P* < 0.001) were observed (**Table 3**).

In addition to DVPs, DGPs and texture features extracted from the dose were analyzed. For the whole rectum, most of the top 10 features (60%) with statistical significance (*P* < 0.001) were DGPs from the low-dose levels (e.g., absolute/relative area, perimeter). For the anterior rectum, the top 10 features were DGPs and textures, most of which were not statistically significant (80%). For the posterior rectum, the most statistically significant top 10 features were DVPs ranging from D_1.0cc_ to D_2.5cc_. The discriminative capabilities of these top-ranked features seemed inconsistent. Those from the posterior rectal wall were more discriminative than those from the whole and anterior rectum (**Figure 6**), suggesting that the rectal insertion of *Kushen Ningjiao* exhibited a dominating dose–volume effect on the posterior rectum, which was found to be predictive of rectal bleeding in previous studies (55, 56). The findings of the present study can serve as the first step toward the ultimate clinical endpoint, that is, unveiling the correlations between doses delivered on different rectal zones and radiation toxicity, as well as confirming whether the proposed rectal protection regimen can finally translate into clinical gains.

The pathogenesis of radiation proctitis has yet to be completely elucidated. However, radiobiological studies showed that excessive radiation to the rectum might cause damage in intestinal crypt stem cells, resulting in crypt involution, mucosal injury, and exposure of the underlying lamina propria to luminal bacteria. These effects cause an acute inflammatory response involving T lymphocytes, macrophages, and neutrophils (57). Giving more protection to the posterior wall may allow the necrotic or fibrotic cells to proliferate, regenerate, and crawl along the rectal wall to renew and replace damaged cells on the anterior rectal wall (12). However, one limitation of the present study was the short follow-up. Hence, further follow-up is required to confirm the gains achieved by rectal insertion of *Kushen Ningjiao*, especially for chronic radiation proctitis, which can develop between 3 months after the radiation therapy to many years later (57, 58). Another limitation was the potential gel migration. The entire treatment process lasted ~25 min, including the rectal insertion of *Kushen Ningjiao* (lithotomy position, ~1 min), CT scanning (maintaining lithotomy position, ~5 min), contouring and treatment planning (~10 min), and treatment (maintaining lithotomy position, ~10 min). The inserted gel was expected to have minor motion or migration because the patients were required to keep the same lithotomy position and reduce movement. However, the actual gel migration is still unclear, and quantifying such motion is difficult, if not impossible. The associated dosimetric impact introduced by the potential gel migration should be noted.

## Conclusion

In summary, this study comprehensively evaluated the dosimetric effects of the use of *Kushen Ningjiao* for rectum protection *via* discriminative modeling of the deformable accumulative dose. A significant dosimetric impact was found on the dose–volume parameters D_1.0cc_–D_2.5cc_ extracted from the posterior rectal wall. Whether this dosimetric increase can eventually translate into a clinical gain still requires further long-term follow-up and more clinical data for confirmation.

## Data Availability Statement

The original contributions presented in the study are included in the article/supplementary material. Further inquiries can be directed to the corresponding author.

## Ethics Statement

Written informed consent was obtained from the individual(s) for the publication of any potentially identifiable images or data included in this article.

## Author Contributions

XW, BZ, and QH: Conceptualization, Design of methodology, Development and implement of models, Original drafting. FH: Data curation and preprocessing. HM: Experimental results analysis, Draft reviewing. YK: Contouring and decide the distribution of the treatment dose. ZD, SZ, YZ, and XT: Data collection and perform brachytherapy treatment planning. XZ: Conceptualization, Design of methodology, Review and editing. All authors contributed to the article and approved the submitted version.

## Funding

This work is supported in part by the National Natural Science Foundation of China (81874216), the Guangdong Provincial Science and Technology Department of Self-financing Projects (2017ZC0165), the Ministry of Education Industry-Academic Cooperation Project (201902119002), the Guangdong medical scientific research foundation (A2019196).

## Conflict of Interest

The authors declare that the research was conducted in the absence of any commercial or financial relationships that could be construed as a potential conflict of interest.
